# Mini-Review of the New Therapeutic Possibilities in Asherman Syndrome—Where Are We after One Hundred and Twenty-Six Years?

**DOI:** 10.3390/diagnostics10090706

**Published:** 2020-09-17

**Authors:** Bogdan Doroftei, Ana-Maria Dabuleanu, Ovidiu-Dumitru Ilie, Radu Maftei, Emil Anton, Gabriela Simionescu, Theodor Matei, Theodora Armeanu

**Affiliations:** 1 Faculty of Medicine, University of Medicine and Pharmacy “Grigore T. Popa”, University Street, No. 16, 700115 Iasi, Romania; bogdandoroftei@gmail.com (B.D.); dabu_93@yahoo.com (A.-M.D.); dr.radu.maftei@gmail.com (R.M.); emil.anton@yahoo.com (E.A); gabi.ginecologie@gmail.com (G.S.); 2 Clinical Hospital of Obstetrics and Gynecology “Cuza Voda”, Cuza Voda Street, No. 34, 700038 Iasi, Romania; matei.theodor1@gmail.com (T.M.); theodoraarmeanu@yahoo.com (T.A.); 3 Origyn Fertility Center, Palace Street, No. 3C, 700032 Iasi, Romania; 4 Department of Research, Faculty of Biology, Alexandru Ioan Cuza University, Carol I Avenue, No. 20A, 700505 Iasi, Romania

**Keywords:** Asherman syndrome, intrauterine adhesions, intrauterine synechiae, hysteroscopy

## Abstract

Asherman syndrome is a multifaceted condition describing the partial or complete removal of the uterine cavity and/or cervical canal. It is a highly debatable topic because of its pronounced influence on both reproductive outcomes and gynaecologic symptoms. The latest reports demonstrated that trauma to the endometrium is the main cause of intrauterine adhesion formation. Left untreated, such adhesions gradually lead to a range of repercussions ranging from mild to severe. Considering the lack of non-invasive approaches, the advent of hysteroscopy has revolutionized the entire field, being otherwise considered the most efficient tool offering new directions and amplifying the chances of treating the Asherman syndrome.

## 1. Introduction. Our Current Knowledge about the Asherman Syndrome

One hundred and twenty-six years have passed since the pioneering work of Heinrich Fritsch. He described the case of a patient that had developed secondary amenorrhea following a posttraumatic curettage [1]. Since that point, after more than three decades, additional information is provided. Bass reports 20 cases of cervical obstruction from a cohort consisting of 1500 patients who had undergone induce abortion [2]. Subsequently, Stamer reviews 37 cases from the literature and adds its own 24 cases concerning intrauterine adhesions (IUAs) associated with the gravid uterus [3].

The Israeli gynecologist Joseph G. Asherman made, in 1948, a complete description of the syndrome that bears his name. Having as support previous observation and evidence, he identifies this pathology in 29 women who present amenorrhea with stenosis of the internal cervical ostium. These initial observations have led Asherman to speculate that such a condition could be the consequence of endometrium trauma. Another case series of IUAs are communicated by the same author in the years that followed involving the uterine cavity with clear flaws observed during hysterography. Therefore, he publishes several manuscripts describing all particularities through which Asherman syndrome (AS) is recognized [4,5].

Although the initial tendency was to establish the prevalence and etiopathology of AS, attention shifted to diagnosis, treatment, and outcome once our understanding regarding the underlying mechanism had been improved [4]. Despite the best efforts, and concomitantly with the wide usage of diagnostic and operative hysteroscopy, the management of AS still remains challenging and intriguing after all this time [6].

Even though studies aiming to delineate distinct particularities of AS were conducted, on fewer occasions the authors offered a detailed documentation. Therefore, comprehensive reports have been produced in South America, the US, Japan, Denmark, France, Greece, and Israel [7]. Koch’s bacillus, blood flukes, surgical interventions, or having a physiological substrate [8] are amongst the main factors associated with IUA formation [4,9].

However, there may be discrepancies between definitions and terminology. Some authors prefer AS to describe patients whose conditions include amenorrhea, surgical scars, or recently obliterated/gravid uterus. On the other hand, IUAs are considered to be more appropriate. However, it does not include those patients suffering from endometrial surface deficiencies without synechiae bridges between the uterine walls [10]. Hanstede et al. [11] appreciate the presence of symptomatology to be the boundary between AS and IUAs.

Although the Asherman’s syndrome was documented more than a century ago and despite the numerous preventive approaches designed, a reliable non-invasive technique to avoid the re-occurrence of adhesions need to be proposed and tested. On the positive side, many pre-, intra-, and post-operative methodologies exist, which can be safely used aiming to improve the surgical outcome(s) in cases of women who suffer from severe AS. These include: hysteroscopy [4], which is considered the gold standard or second-look office hysteroscopy [12,13], transvaginal ultrasound (TVS) [14], or laparoscopy-guided/transabdominal ultrasound (TAS)-directed hysteroscopy dedicated in repairing intrauterine synechiae [15].

Moreover, techniques such as hormonal manipulation with estrogen [16], foreign agents dedicated to increasing the blood flow [17], or physical constructs specifically designed for the uterine cavity [18,19] fits within the current preventive approaches against IUAs. The applicability of all these methods combined could be exponential with high chances of success, and implicitly positive reproductive outcomes, but no report is available in the literature.

Thus, the present mini-review aims to discuss all evidence with a special focus on management-therapy/treatment, in parallel concerning AS’s prevalence-incidence, classification, implications, diagnostic, and risk factors.

### 1.1. Classification. Is There a Clear Classification Panel for Asherman Syndrome?

There have been numerous attempts at classification (Table 1), Toaff and Ballas proposing the first model back in 1978 [20]. Congruent with their findings by hysterosalpingography, IUAs were tabulated into four groups. Therefore, the advent of hysteroscopy promoted a series of investigations depending on other criteria [21,22,23].

Thirty-two years ago, The American Fertility Society designed an algorithm for the classification of the IUAs based on the menstrual history and having as support hysteroscopic and hysterosalpingographic data [24]. In parallel, March et al. [22] introduces their model the same year. However, the model propose by March has been catalogued as insufficient, but it is further applicable for its simplicity [5,25].

From another point of view, the European Society of Hysteroscopy (ESH) and European Society of Gynecological Endoscopy (ESGE) [26] approved the model design by Wamsteker at the Hysteroscopy Training Center [27]. Unfortunately, both these schemes have failed because of their complexity and difficulty. This concept also applies to the scoring system proposed by Nasr et al. [25]. In terms of good correlation, can offer a more appropriate prognostic in women that suffer from mild or severe AS, but inefficient for those with moderate adhesions.

### 1.2. Incidence. How Common the Asherman Syndrome Actually Is?

The incidence of IUAs is difficult to ascertain because few studies assess the occurrence of adhesion formation. Considering the higher susceptibility to injury of the pregnant uterus between the 2nd and 4th weeks post-partum, we illustrated in Table 2 the most common risk factors and those studies aiming to establish the incidence of IUAs. However, these figures are also influenced by the type of intervention, without being strictly related to the status of the uterus.

A recent systematic review and meta-analysis aimed to reveal the prevalence of IUAs in a cohort consisting of nine hundred and twelve/one thousand seven hundred and seventy women. A pooled prevalence of 19.1% towards IUAs was charter in one hundred and eighty-three women. A pooled prevalence of 58.1%, 28.2%, and 13.7% was also reported concerning the risk of extended IUAs with all three degrees of severity in one hundred and twenty-four women (67.8%). These values suggest that D and C could be behind these associations. From six hundred and seventy-five women, one hundred and fifty cases of congenital and acquired IUAs were registered, with a pooled prevalence of 22.4%. Conservative, medical, or surgical management prove to be equally efficient in cases of miscarriage despite the limited number of women included [29].

Another study carried out by the same author discloses a prevalence of IUAs of 21.2% in women ranging from moderate to severe in 48% during the first trimester after surgical Termination Of Pregnancy (TOP). The prevalence of IUAs in women after the second-trimester TOP evaluated by hysterosalpingography was 16.2% by intra-amniotic prostaglandin [30]. Hooker et al. [31] unravel in hysteroscopically evaluated women an IUA prevalence of 22.4%. In the second study that falls within the scope, the rates between D and C and hysteroscopic resection were from 30% to 13%, whereas in the context of incomplete evacuations after D and C and hysteroscopic resection were from 29% to 1%.

Previously, an incidence of the fibrotic tissue ranging from 10% up to 30.6% after one or multiple curettages was shown. These figures had been obtained after a series of investigations by hysterosalpingography and hysteroscopic evaluation [41,42]. A relatively high incidence (15%) after suction D and C in patients who had suffered Spontaneous ABortion (SAB) but with the normal cavity prior documented was presented [28]. Furthermore, a significant percentage (70%) of patients with severe AS had undergone instrumentation during the postpartum period. However, in those with a mild condition, figures were even higher (80–90%) during the first trimester of pregnancy [11].

From a cohort of three hundred and twenty-six women, only three perioperative complications were identified, and zero cases of incomplete retained Product Of Conception (POC) removal. On follow-up hysteroscopy, only in four cases out of ninety-six women were noted IUAs. Out of one hundred and twenty patients, ninety-one women achieved pregnancy [32]. In Denmark, there was a registered decline in surgical termination between 2001 to 2017 from 87% to 36%, but this proved insufficient in 4%, leading to curettage [43].

There are also controversies concerning IUA recurrence following hysteroscopic myomectomy. Yu et al. [34] emphasize an incidence of IUAs of 24% following hysteroscopic resection of the uterine septum. On the other hand, March [10] underlined situations where authors reported an incidence between 30% and 45%. A recent prospective study discusses the high chance of IUA reformation (22%) after abdominal myomectomy procedures [36].

There are numerous other procedures that may promote IUA formation. Thus, case stories with reference to B-Lynch sutures, surgical treatment of Müllerian anomalies, and embolization of the uterus have also been discussed on several occasions [34,44,45].

Based on the aspects presented in this section, the incidence/prevalence of AS could be explained by several factors, such as (I) the number of abortions performed (il) legally; (II) genital tuberculosis or puerperal infections; (III) awareness of the clinicians; (IV) criteria for IUA diagnosis; and (V) the instruments used to conduct the respective procedure [46].

### 1.3. Pathophysiology. The Interplay between the Risk Factors and the Current Methodologies Associated with Intrauterine Adhesions Formation and Re-Occurrence

From a mechanical point of view, evaluation through the electron microscopy of endometrial ghiandolar cells revealed important sub-cellular anomalies in women with severe AS. Among the most severe modifications is the lack of ribosome biogenesis through which can be explained the ATP depletion and the subsequent impairment of ionic pumps. Therefore, the activation of oncosis, and concomitantly with the associated hypoxia symptomatology should be a topic of great interest considering the disruption of homeostasis. The expression of vascular endothelial growth factor (VEGF) and the score of MVD (micro-vessel density) significantly change after treatment, as well as in the control group [47].

Continuing with this concept, such biological processes would reflect the reliability of the procedure. Following a transcervical resection, an abnormal expression of several growth factors’ (transforming growth factor beta 1 (TGF-β1), platelet-derived growth factor (PDGF), and basic fibroblast growth factor (bFGF)) was noted within the related areas, which is associated with adhesion-related cytokine activation [48].

Another explanation of the pro-inflammatory cascade triggered can be attributed to the disturbance of the human microbiome. It has been already highlighted that fluctuations of the microbial communities that populate every individual can gradually cause the loss of eubiosis, resulting in a significant predisposition towards psychiatric and neurodegenerative disorders. During intrauterine life, the risk is also high. Beyond the risk of a possible neurological disorder, it has also been discussed about the repercussions that C-sections had on an infant’s microbiome, such as metabolic or cardiovascular problems the baby could suffer from in the future [49].

Another example is represented by the patients with IUAs where significant differences at the phylum and genus level were observed (more precisely, a significant reduction in Firmicutes and *Lactobacillus*, to the detriment of Actinobacteria, *Gardnerella*, and Prevotella [50]. The presence of, and subsequent infections caused by, Mycobacterium tuberculosis is also detrimental. Not only is it positively associated with the recurrence of AS, but also with the severity of IUAs, and poor prognosis [7,51]. It has been hypothesized that *Schistosoma sp.* could be involved in AS development, but with the mention that this prognosis should be excluded from those parts of the world where it is endemic [52].

It is certain that both commensal and pathogenic entities could independently or in a collaborative manner influence the outcome following surgical intervention, having branched and pronounced consequences upon the patients.

### 1.4. Diagnosis. What Is Currently Used to Diagnose Asherman Syndrome?

During the last couple of decades, technologies have been designed (Table 3) dedicated to exploring the uterine cavity that revolutionized the diagnosis of AS. Although the suspicion of AS could exist depending on the symptoms, there are situations when the patients are asymptomatic. It should be mentioned that, in Table 3, we included only those studies that reunited cohorts >100 patients. The search was performed using several key words, such as “adhesions”, “synechiae”, “sensitivity”, “specificity”, “intrauterine abnormalities”, and “intrauterine pathology”. We excluded those studies that could not be accessed, or the number of patients was not mentioned.

Retrospectively, hysterosalpingography (HSG) was the first-line tool consecrate to confirm the presence of IUAs. It is still considered by many gynecologists valid for detecting filling abnormalities. It is cost effective, and it possesses a sensitivity between 0% and ~100% and >30% and 100% specificity. In terms of imaging, both HSG and sonohysterography (SHG) were equally sensitive [10]. HSG was found to be as accurate as hysteroscopy despite the fact that the nature of the filling defects was detected by hysteroscopy as has been demonstrated in a retrospective study of 400 patients [71]. It was also shown that approximately 38.3% of HSG examinations were false-positive [59]. Among 65 women from which five had hysteroscopic evaluation, HSG had a sensitivity of 75% of IUA detections and 50% predictive value when compared to hysteroscopy according to the results of Soares et al. [72].

A recent systematic review and meta-analysis treats saline infusion sonohysterography’s accuracy in detecting intrauterine abnormalities, and it was concluded that it has an 82% up to 99% pooled sensitivity and specificity [73]. Another retrospective study of 149 cases reveals a significant difference between the HSG group and SHG group (50.3% and 81.8%) [54]. Due to its concept, SHG is superior to transvaginal ultrasonography at detecting IUAs [67]. Among 65 infertile women, SHG had a sensitivity of up to 75% and a specificity around 42.9% to HSG [72]. A non-invasive procedure usually applied when HSG is not possible is ultrasonography. It was used in two previous occasions by Conforti et al. [74] and Schlaff and Hurst [14], but both sensitivity and specificity are quite low; sensitivity up to 52% [75], and 11% specificity [67]. 

Despite the fact that transvaginal ultrasound demonstrates a substantially thinner endometrium between the AS group (*n* = 16) and control group (*n* = 50) [76], the accuracy is low [67,75]. However, it is cheaper compared to laparoscopy, with no significant differences concerning IUA incidence [15]. Both unenhanced transvaginal ultrasonography and contrast saline infusion sonohysterography (SIS)have a finite diagnostic capacity [75], near 0% specificity and sensitivity [72], and moderate to high positive and negative predictive values (98% and 43%) [72,75].

Few investigators have used three-dimensional ultrasonography aiming to detect IUAs [67,77]. It even succeeds with a specificity of around 45% [67]. It was found that 3D-SHG has a sensitivity and specificity around 91.1% and 98.8%, respectively [62], preliminary results that have been confirmed by Abou-Salen et al. [78]. Three-dimensional ultrasound was compared with hysteroscopy, confirming a variation that ranges between 16% and 100% based on a series of criteria, as shown in a recent Taiwanese study involving 110 women [79]. There are rare situations when magnetic resonance imaging (MRI) is indeed valuable as a supplementary diagnostic tool [4,43].

Despite all the above and the developments, hysteroscopy is rightly called the gold standard due to the fact that it allows direct real view visualization of the endometrial cavity. Moreover, it can be performed “in office” with minimal discomfort than a blind HSG [4]. Virtual hysteroscopy could play the key role in the future in the diagnosis of IUAs [80].

#### 1.4.1. Treatment for the Asherman Syndrome and the Related Complications?

##### Dilatation and Curettage

Prior to the fulminant ascension that hysteroscopy had, D and C was the method of choice. This gynecological procedure is dedicated in removing tissues from inside the uterus used in the management of first and second trimester miscarriage as well as for TOP. Despite the fact that it was considered a relatively safe and easy-to-perform technique, serious adverse effects may occur. It has been documented that D and C could cause both short-term and long-term complications, such as cervical tears, bleeding, infection and perforation of the uterus, something resulting in a perforation of the bladder or bowel [81,82,83,84]. Congruent with our topic, D and C is known to be a promoter of IUAs, or increases the risk of preterm birth [7,29,85].

Hefler et al. [86] demonstrated that from a large cohort of 5459 nonobstetric patients, in only 1.9% (*n* = 103) of cases intraoperative complications were noted. The authors concluded that the complication rate of D and C is low, retroverted uterus, postmenopausal status, and nulliparity being independent associated risk factors. The proportionality of the success/complications rate can vary. The risk of AS was found to be 30.9% in women after one miscarriage [7] and 25% in women who had D and C in the first 1–4 weeks postpartum [87,88].

In a study of 11,914 women who underwent D and C over a 7-year period, there were only 23 cases in which patients sustained a uterine perforation. Amarin et al. [89] argued that in 22 cases the operator was a trainee and that previous surgery was performed in only two of these 23 cases. The authors suggest that this rate is attributed to the operators’ inexperience.

##### Hysterotomy

Another widely used procedure among women between 40 and 50 years of age is hysterectomy. The removal of the uterus is usually applicable in order to treat many women’s health conditions [90]. This technique was implemented by Reddy and Rock [91], where they reported the case of three patients who had previous unsuccessful hysteroscopic resection of the IUAs. In order to delimit the uterine walls and identify the internal os, a uterine sound was placed in the cavity transcervically, whereas, for the profiling of the corneal areas affected by the scars, a 2.0 nylon suture was made threaded through digitized branch structures (fimbria) to pass into the uterus.

On four distinct occasions, the authors compared laparoscopic, vaginal, and abdominal hysterectomy. It has been concluded that are no significant differences between groups with respect to BMI, parity, intra- or postoperative major, and minor complications. The time was shorter in the vaginal hysterectomy (VH) group (*p* < 0.001), whereas the laparoscopic hysterectomy (LH) group was defined by a significantly low blood loss (*p* < 0.001) and the duration of hospitalization and analgesic (*p* < 0.001). However, in the VH group, a low rate in terms of uterus weight and intra-abdominal surgery (*p* < 0.001) was noted [92].

Analogous, dysfunctional uterine bleeding (DUB) was a common indication for hysterectomy. In relation to time, the minimum blood loss was registered in total laparoscopic hysterectomy (TLH), with significant differences in terms of pain scores using Visual Analogue Score (VAS) among all three groups on day 0 and day 1. In the VH group the lowest score was recorded, while regarding the maximum hospital stay was noted in the TAH group and no differences between VH and TLH [93].

Nagata et al. [94] reviewed the clinical records of 102 patients where TLH and abdominal total hysterectomy (ATH) were performed in 55 and 46 cases, respectively. In the TLH group, a significantly longer total operation time, lesser blood loss, shorter hospital stay and lighter uterine weight were noted than in the ATH group, and with no fluctuating frequency among perioperative complications between these two (3.5% vs. 8.0%).

Among all indications between TLH, abdominal hysterectomy (AH) and LH, TLH is a safe and less invasive approach, especially towards AH (uterus weight, the need for analgetics and hospitalization time with *p* < 0.001), and with high chances of post-operative reconstitution. Even though VH (median age; *p* < 0.001) is faster and more efficient, TLH has the advantage by offering the possibility to view the intra-abdominal situs and to act accordingly in pathological cases [95].

From a total of 12 reports, and 31 cases of hysterotomies, approximately 52% (*n* = 16) of women conceived, and 25.8% (*n* = 8) had term deliveries [7]. Another study describes only three cases of successful restoration, two of them conceiving and having live births [96]. However, it is advisable to use this technique only when the situation requires, and hysteroscopy is inefficient. The patient should be informed and warned that success is not guaranteed [4,97].

##### Hysteroscopic Adhesiolysis

The magnification and the central view of adhesions should be divided by using the tip of the hysteroscope in order to be easy to differentiate. Proximal structures are intricate because of the high risk of uterine perforation. Mechanical and electric cutting settings are used in this context. Caution should be maximal as errors may have unwanted repercussions. There were pros and cons, monopolar diathermia being finally related to fluid overload complications in contrast to bipolar diathermia with saline [4]. Several papers describe the outcome after hysteroscopic adhesiolysis with a success rate that ranges between 75 and 100%. The fertile potential is shaped by a series of exo- and endogenous factors, which directly affects the pregnancy rate (25% up to 76%) and term delivery rate (25% up to 79.7%) [4,43]. Each process has advantages, hysteroscopy being feasible due to the fact it can be accomplished in an outpatient framework. Intraoperative fluoroscopy and transabdominal ultrasonography, or laparoscopy are also efficient alternatives, each one with a higher degree of sensitivity and low degree of error [4,5,43].

A cohort study aiming to study the success rate of hysteroscopic adhesiolysis and the spontaneous recurrence rate of IUAs by reuniting 638 women with AS offered some conclusive results. A first-trimester procedure preceded AS in 58.2% (*n* = 371) of the cases causing IUAs of grades 1–2A, while this figure was 38.1% (*n* = 243) for a postpartum procedure with IUAs of grades 3–5. Hysteroscopic adhesiolysis was applied successfully in 606 women (95%) with the restoration of menstrual blood flow in 97.8%, while IUAs recurred in 27.3% (*n* = 174) [11].

Another large retrospective prospective study performed in order to investigate the prevalence, effectiveness, outcomes, complications and indication of operative hysteroscopy describes a total of 1919 hysteroscopic procedures that were performed as follows: 1829 (95.3%) diagnostic and 90 (4.7%) operative hysteroscopies. The most common operative procedure was applied for fibroid polypectomy (34.4%), followed by transcervical resection of the endometrium (25.6%) and endometrial polypectomy (17.8%). Menorrhagia (63.33%), recurrent miscarriages (10.00%), and primary infertility (5.66%) were considered as indications, with 73.33% and 26.67%, respectively, of the cases being treated under general and spinal anesthesia. In 48.4% and 50% fibroids and polyps were 3 to 4 cm and in 35.5% the fibroids were > 4 cm. Furthermore, in 60.3% of the patients who suffered from heavy bleeding, 44% conceived, 2.2% had excessive fluid absorption and 1.1% had uterine perforation [98].

Another retrospective analysis of only 24 patients was conducted aiming to evaluate postoperative blunt adhesiolysis and sharp adhesiolysis for women with menstrual disorders, pain, or infertility resulting from IUAs. According to the March criteria, 83% of patients presented with amenorrhea or oligomenorrhea, 67% had severe adhesions, 46% moderate, and 4% minimal. Following the conclusion of this study, 95% improved their menstrual flow, 92% were characterized by a relief of dysmenorrhea, and 46% of infertile patients achieved pregnancy or already delivered one [13].

In a prospective randomized trial, 71 patients were divided into two groups with a similar design; three office hysteroscopies with an IntraUterine Device (IUD) insertion and 2 months estrogen and P therapy for severe IUAs. At the end of the present trial, spontaneous pregnancy and live birth rates for both groups were as follows: 47.2% and 30%, 28% and 20%, respectively [12]. A Chinese retrospective observation study with double the number of patients compared to the previous one showed that the general pregnancy rate was 71.5%, while the live birth rate was 53.0% in women treated for IUAs. The pregnancy rate was higher in the amenorrhea and recurrent miscarriage groups compared with normal menses and infertility groups, logistic regression showing that the second-look time interval, pregnancy history and times of operation to relieve adhesions were positively associated with the pregnancy rate, while age and the second-look interval with the live birth rates [99]. Taking into consideration the number of patients initially included, the pregnancy rate oscillates around the middle (42.8%) according to a case report series, and with a live birth rate of around 32.1%. From a total of nine patients with live births, only one Caesarean hysterectomy for placenta accreta and one hypogastric arteries ligation for severe haemorrhage and placenta accreta were conducted [100].

Zikopoulos et al. [101] reported a study on a 10-year experience concerning the treatment of subfertile women with IUAs by using a resectoscope or the Versapoint system that were divided into three categories depending on the stage: stage I (*n* = 6), stage II (*n* = 25) and stage III (*n* = 15). Twenty-one underwent adhesiolysis through resectoscope and twenty-six Versapoint. The overall procedures had a success rate of 93.5% after the first attempt, in 92.9% of the cases of oligo/amenorrhoea at presentation restoration being performed successfully in 9/9 for Versapoint and 4/5 resectoscope. The overall live delivery rates were 33.3%, 44.4%, and 46.7%, similar percentages being obtained in patients with no additional infertility factors (Versapoint—71.7% and resectoscope—60%). Half of the gestations (50%, *n* = 10) have ended up with a preterm delivery, from which in two women who delivered, a hysterectomy was conducted due to placenta accreta.

##### Stem Cell Therapy

Another, much more efficient, approach is that with stem cells due to their potential of multiplication as a stem cell in an undifferentiated form and to mature and differentiate, but also to produce various other types, such as totipotent, pluripotent, multipotent and unipotent cells [102].

In both experimental models [103,104,105,106,107] and humans [108,109,110,111] the regeneration of endometrium through a stem cell approach has been evaluated. Mesenchymal, bone-marrow-derived stem cells and menstrual blood-derived stromal cells were investigated in this context. For this, distinct methods such as transmyometrial administration to the subendometrial area [109], direct installation of stromal cells in the uterine cavity after endometrial scratching [111], and infusion in spiral arterioles through catheters [110] have been put in application, even stem cells arranged in spheroids for a rat model [107]. Thus, the first results were not long in coming. Five out of six women regained their menstruation once again, from those attaining endometrial thickness and regular menses conceiving not long after, one female even giving birth or had an ongoing pregnancy [109,110,111].

In Table 4, we summarized studies on regenerative medicine and cell therapy having as scope treating injured endometrium or repairing thin endometrium and the related fertility rate effects.

#### 1.4.2. Preventive Approaches of IUAs Occurrence

##### Intrauterine Balloon Stent

Is a mechanical device designed by Cook Medical Inc, Bloomington, USA, made of silicon with a triangular shape that molds to the shape of the uterine cavity and dedicated in preventing adhesions recurrence.

It was recently revealed a bacterial colonization following balloon uterine stent placement for 1 month. Of the 68 women initially included, eight were excluded, being demonstrated a bacterial colonization before surgery in contrast to nine women (30.0%) after 1 month in the stent group (10.0%). However, in the control group, 4 (13.3%) and 10 (33.3%) women had microscopic entities detected prior to and after 1 month after surgery [121].

A recent comparative analysis aimed to evaluate the efficacy of both intrauterine balloon and intrauterine contraceptive devices (IUDs) for the prevention of IUA reformation after hysteroscopic adhesiolysis. Only 162 continued the procedure because 39 cases dropped out. The results obtained by Lin et al. [122] in terms of efficiency are almost identical to the median adhesion score reduction, which was 7 for the balloon and IUD group, while the adhesion reformation rate was between 30 and 35% for the balloon and IUD group. Another study showed that a small number (*n* = 19; 25%) women present IUAs reformation after hysteroscopic adhesiolysis when an intrauterine suitable balloon was used. The figures were higher in the Foley balloon (*n* = 26; 35.1%) [123].

Pisani et al. [124] described the case of a 43-year-old woman with an initial diagnostic for secondary amenorrhea. Her clinical history included an SAB followed by D and C and a hysteroscopic adhesiolysis for AS. The diagnostic hysteroscopy revealed a hematometra and an obliterated uterine cavity with multiple adhesions. They performed an ultrasound-guided hysteroscopic adhesiolysis and a balloon stent was inserted. The catheter was kept for five consecutive days and antibiotic coverage. The additional diagnostic hysteroscopy performed after 1 and 2 months showed a regular uterine cavity without the reformation of IUAs.

The pregnancy rate among 1240 women in which an intrauterine stent was used is 61.6% and with a relatively low SAB rate of 15.6% [10]. Another retrospective cohort study of 107 patients aimed to compare the efficiency of IUD, hyaluronic acid, and intrauterine balloon stent resulting in a significant reduction of adhesion recurrence rate [125].

##### The Foley Catheter

The Foley catheter was among the first devices dedicated to separating the uterine walls to prevent the recurrence of IUAs. Seventeen years ago, Orhue et al. [126] assessed the use of a Foley catheter balloon and IUD as a possible adjuvant treatment. In the initial 4-year period, patients with IUAs were treated initially with an IUD inserted for 12 weeks after adhesiolysis (*n* = 51). The subsequent 4 years, a Foley balloon was used for almost 1 and a half week after adhesiolysis. While in the Foley group a significant percentage (81.4%; *n* = 59) of the patients had their menstruation restored in contrast with 62.7% in the IUD group. A one-time technique was appraising in a study consisting of a group of 25 cases with moderate and severe adhesions. A fresh amnion graft was inserted over a Foley’s catheter balloon into the uterus for 14 days after the hysteroscopic intervention. Only two cases with potential damage as a consequence of this approach, but an overall improvement in terms of length and no adhesion reformation were noted [127].

##### Hyaluronic Acid

Hyaluronic acid is one of the most widespread component in tissues, participating in a plethora of biological functions such as cell proliferation and migration. There is an elevating trend in the literature where products derived from hyaluronic acid have been adopted in this field for preventing IUAs [128,129,130].

Even though the auto-cross-linked hyaluronic acid (ACP) gel significantly reduces the incidence and severity of IUAs, it does not suppress the possible formation afterwards, as shown in a recent randomized controlled trial. There were 152 women included in this study who had suffered a miscarriage less than 14 weeks ago, and with at least one D and C for miscarriage or TOP. From the total number, only 149 outcomes were available: two groups divided unequally (77 in the intervention group and 72 in the control group). From the total number, IUAs were observed in ten (13%), and twenty-two patients (30.6%), respectively [42].

Another randomized controlled trial aims to evaluate the efficiency of a new cross-linked hyaluronan (NCH) gel in 300 women in order to reduce the formation of IUAs after D and C. It should be mentioned that the authors lost the records of 26 women who were therefore excluded from the study. From the rest of the 274 women, data were available for 137 in each group post-operative. In both groups, IUA formation was observed in 13 and 33 women (9.5% and 24.1%). Moreover, the NCH gel group has registered significantly low scores in terms of the type of adhesion, cumulative adhesions, menstrual pattern, and extent of intrauterine cavity involved. As well in the NCH gel group, the proportion of patients with moderate to severe IUAs was significantly lower compared to the control group (1 case [0.7%] vs. 16 cases [11.7%]) [131].

We have identified in the literature three meta-analyses in which the authors discuss the usage of hyaluronic acid to prevent IUAs after intrauterine operations, hysteroscopic adhesiolysis, and miscarriage. From a total of seven randomized clinical trials with 952 patients, the usage of hyaluronic acid gel significantly reduced the incidence of IUAs. The efficiency of the hyaluronic gel was independent to the type of procedure, these figures being as follows: abortion, hysteroscopy, primary disorders/diseases such as abortion, IUA, submucosal myoma, endometrial polyps or mediastinum uterus. Furthermore, the hyaluronic gel improved pregnancy rates [132].

Intriguingly, from a total of six articles and 394 patients subjected to hysteroscopic adhesiolysis, there were no statistically significant differences among women who recieved hyaluronic acid gel in terms of scores towards IUAs, neither between the same groups on the recurrence. There was a slight reduction rate of IUA recurrence in randomized controlled trials, with no significant effect in terms of pregnancy rate after IUA separation [133].

Another meta-analysis conducted by Fei et al. [134] brought some interesting results. Hyaluronic acid gel reduced IUA scores after miscarriage and the incidence of post-operative IUAs after miscarriage. A subgroup analysis revealed a reduction of incidence towards moderate and severe IUAs after miscarriage, but with no effect of mild IUAs. Finally, it improved the pregnancy rate after miscarriage.

Conflicting results were also obtained after the investigation of hyaluronic acid gel or polyethylene oxide-sodium carboxymethylcellulose in order to prevent IUAs and the authors concluded that there is a lack of reproducible evidence [135]. There is no study that favors the use of any gel barrier following operative hysteroscopy for key indices such as the live birth, clinical pregnancy or miscarriage, just a decrease incidence of de novo adhesions at second-look hysteroscopy [136].

Taskin et al. [39] demonstrate an elevating rate of IUAs after the resection of single and multiple fibroids (31.3% and 45.5%), while the incidence after resection of the uterine septum was around 12% [137]. Moreover, Mazzon et al. [138] deepen this spectrum and indicate a low frequency of IUAs after the myomectomy of monopolar current and cold knife resection.

Nor through the oral estrogen administration have better results been obtained [137,139,140]. A recent systematic review and meta-analysis conducted by Chang et al. [141] also exposed the relatively low efficiency of estrogen therapy, the quality of evidence ranging from moderate to very low with only a short-term or negative effect.

##### Intrauterine Device

This T-shaped intrauterine device usually used to prevent a pregnancy has brought controversies around its use, some authors even reporting satisfactory results [142,143]. Vesce et al. [144] observed good results in a group consisting of 48 women with functional amenorrhea after the use of a copper IUD, shortly thereafter a significant number of women regaining their regular menses. Despite this evidence, some believe that inflammatory-related factors from copper could in fact aggravate the pre-existing endometrial damage [145]. In women randomized to receive an IUD, estrogen, or no treatment, no differences have been noted by Touguc [137]. From the Levonorgestrel-releasing IUD, T-shaped IUD, and Lipples loop, the last one was considered to be more adequate thanks to its trapezoidal shape because of either the Levonorgestrel effect on the endometrium or the device was too small [10].

Nevertheless, these procedures are not entirely risk free, and complications may occur, for example, after adhesiolysis [4] or obstetric risks such as reduced fetal weight or related to the placenta. Only one case could be identified in literature where the hazardous repercussions upon the newborn from a mother that had an IUA are described. An increased incidence of preterm deliveries, retained placenta (10.7%), and a significantly reduced weight associated with IUAs was shown, but the main disadvantage was the inclusion of only 14 cases and 42 controls [146]. Roy et al. [140] and Zikopoulos et al. [101] had similar approaches in their study, concerning the live delivery rates. Only recently have cases of placenta accreta been described [147]. This severe pregnancy condition has been noted in 8% of the women after treatment for AS [148], Friedman even describing three cases of placenta accrete, paper-thin uterine fundus, and uterine sacculation after the treatment of IUAs [96].

It has been revealed by three distinct teams of researchers that menstruation dysfunction, pregnancy rate, live birth rate, adhesion grade, uterine length, complications, reproductive outcome, or miscarriage were significantly lower in patients who receive Interceed plus an IUD, amnion graft, and silicone sheet are reliable adjuvant therapies for AS [149,150,151].

Adhesions barriers, hormonal treatment, uterine stents and IUDs have proven efficient alternatives, yet additional comparative reports are needed. Endometrial stem cells represent a novel approach that offers the possibility to deepen the psychopathology spectrum, which would constitute an entire area for future research. To summarize the above, we decided to offer a conclusive image regarding the main strategies for treating and preventing AS that can be divided into four main steps (Table 5).

## 2. Conclusions

Based on the literature that has been discussed throughout this mini-review, it can be concluded that Asherman syndrome is a worldwide disease with a high impact on female reproductive potential. A plethora of techniques have been designed throughout these last two decades. It is certain that comprehensive approaches are imperative during the early stages of the disease in order to improve the outcome, even for those women who conceived after AS treatment. The introduction of hysteroscopy had an exponential impact, reflected by the fertility outcome and success rate, but the management of moderate and severe disease still represents a challenge. From another point of view, molecular and cellular research is mandatory, but at the same time, the intestinal microflora should not be omitted because its implications are much more complex than are perceived at first sight.

## Figures and Tables

**Table 1:** Classification models for the Asherman syndrome.

**Toaff and Ballas 1978 [20]**			
**Type**	Condition		
Type 1	Morphological abnormalities at the level of the cervical canal devoid of physiological adhesions.		
Type 2	Narrow at the level of the cervical canal associated with a complete obstruction devoid of physiological adhesions.		
Type 3	Numerous fine adhesions at the level of the cervical canal (isthmic region).		
Type 4	Supra isthmic diaphragm producing an entire partition of the prime cavity from its lower section.		
Type 5	Morphological abnormalities at the level of the cervical canal presenting physiological adhesions.		
**March (1978) [22]**			
Mild	Filmy physiological adhesions retaining around ¼ of the uterine cavity. Ostial regions and upper fundus of the uterus insignificant implicated or apparent.		
Moderate	¼ up to ¾ of the cavity implicated. Ostial regions and upper fundus of the uterus partially implicated without any attachment of the uterine walls.		
Severe	Better than ¾ of the cavity implicated. Obstruction of both ostial regions and upper fundus of the uterus with further attachment of the uterine walls.		
**American Fertility Society (1988) [24]**			
Extent of cavity implicated	Less than one third 1	One third to two thirds 2	More than two thirds 4
Type of adhesions	Filmy 1	Filmy and dense 2	Dense 4
Menstrual pattern	Normal 0	Hyper-amenorrhea 2	Amenorrhea 4
Prognostic classification (HSG and hysteroscopy score)	Stage I (Mild) 1–4 Stage II (Moderate) 5–8 Stage III (Severe) 9–12		
**European Society of Gynecological Endoscopy (ESGE) (1995) [26]**			
I	Fine and filmy adhesions		
II/IIa	Sole compact adhesions/Obstruction with adhesions just at the region of the cervical canal		
III	Numerous compact adhesions		
IV	Broad compact adhesions associated with the partial obstruction of the uterine cavity		
Va/Vb	Broad endometrial and fibrotic scarring in relation with grade I or II adhesions/Wide endometrial and fibrotic scarring		

**Table 2:** Incidence-prevalence and risk factors associated with intrauterine adhesion (IUA) formation.

Condition	Procedure	Incidence	Reference
Gravid			
SAB	Suction D and C	15% 19%	[28,29]
First trimester TOP		21%	[30]
Retained POC		30%	[31]
Retained POC	Hysteroscopic resection	6% 13% 19%	[31,32,33]
**Gynaecologic**			
Septum	Hysteroscopic septum resection (bipolar)	24%	[34]
Fibroids	Hysteroscopic myomectomy (bipolar) Abdominal myomectomy	8% 22%	[35,36]
**Risk factors**			
**Common factors**	**Frequency (number of patients)**		**Reference**
Miscarriage curettage Postpartum curettage Diagnostic curettage C-section Trophoblastic disease evacuation Infection (Genital tuberculosis) Abdominal myomectomy	66.7% (1237) 21.5% (400) 1.6% (30) 2% (38) 0.6% (11) 4% (74) 1.3% (24)		[7]
Müllerian duct malformation	16% (7)		[37]
Uterine artery embolization	14% (7)		[38]
Hysteroscopic surgery: Myomectomy (single/multiple myoma) Metroplasty Endometrial ablation	31.3% (10)/45.5 (9) 6% (1) 36.4% (8)		[39]
Insertion of IUD	0.2% (3)		[7]
Uterine compressive sutures for post-partum haomorrhage	18.5% (5)		[40]

SAB—Spontaneous ABortion; TOP—Termination Of Pregnancy; POC—Product Of Conception; C-section—Caesarean section; IUD—IntraUterine Device.

**Table 3:** Sensitivity and specificity of each technique used for intrauterine pathology diagnosis.

Technique	Comparation and Number of Patients	Sensitivity	Specificity	Reference
**Hysterosalpingography** **(HGS)**	HGS vs. HS (*n* = 336)	98.0%	34.9%	[53]
	HGS vs. SHG (*n* = 149)	58.2%	25.6%	[54]
	HGS vs. HS (*n* = 216)	80.3%	70.1%	[55]
	HGS vs. HS (*n* = 120)	74.6%	79.5%	[56]
	HGS vs. HS (*n* = 100)	50%	98.1%	[57]
	HGS and SHG vs. HS (*n* = 149)	58.1%	74.4%	[58]
	HGS vs. HS (*n* = 106)	79%	60%	[59]
	HGS vs. HS (*n* = 359)	21.56%	83.76%	[60]
	HGS vs. HS (*n* = 296)	75.21%	41.14%	[61]
	3-DHS vs. HS (*n* = 124)	91.9%	98.8%	[62]
**Sonohysterography (SHG)**	Saline SHG vs. HGS (*n* = 101)	62.5%	98.9%	[63]
	3D SHS vs. HS (*n* = 141)	97%	100%	[64]
	SHG vs. HS (*n* = 122)	12.8%	97.3%	[65]
	3D gel installation SHG and 2D gel installation SHG (*n* = 110)	98%	94%	[66]
**Ultrasonography (US)**	3D US vs. 2D and 3D SHG (*n* = 209)	98%	100%	[67]
	Transvaginal US (*n* = 78)	100%	96.3%	[68]
	Transvaginal US (*n* = 77)	91%	100%	[69]
	Transvaginal HS (*n* = 60)	92.7%	78.6%	[70]

**Table 4:** Summary of the actions in the field of regenerative medicine and cell therapy.

Type of Stem Cell and Year	Model of Study	Main Observations	Reference
MSCs (2014)	Rat	The regeneration of endometrium is stimulated when MSCs are added to estrogen.	[103]
BMDSCs (2014)	Mice	After the transplant, the rate of fertility has been improved in AS mice, thus indicating a functional role of BMDSC in uterine regeneration.	[104]
hAMSCs (2017)	Rat	A boosted endometrial regeneration was noted following the transplantation of hAMSC in injury-induced IUAs in rat, probably due to immunomodulatory reactivity.	[106]
Autologous adult stem cells (2011)	Human	Endometrial angiogenic stem cells derived from autologous adult stem cells could be used in reproducing damage to the endometrium that do not respond to prevalent therapy for AS; the endometrium thickness is also increased.	[108]
autologous SCs (2014)	Human	A key finding following the transplantation of autologous stem cells in endometrial regeneration was the menstrual reconstruction in 5 out of 6 cases evaluated.	[109]
Autologous CD133 + BMDSCs (2016)	Human	During the first twelve weeks after CD133^+^ BMDSCs therapy a growth in the congestion of mature vessel, and the severity and period of menses respectively were noted. In as, the thickness of endometrium increased from 4.3 mm to 6.7 mm.	[110]
menSCs (2016)	Human	Endometrial thickness in women with severe AS have been improved considerable following the transplantation of Autologous menSCs.	[111]
BMDSCs (2004)	Human	The results of this study revealed that the origin of endometrial cells can be bone marrow cells, and the authors suggest that nonuterine stem cells participate in endometrial tissue regeneration.	[112]
BMSCs (2016)	Rat	The transplantation of bone marrow stem cells (BMSCs) had a pronounced regenerative effect upon endometrium, probably by promoting the expression of estrogen (ER) and progesterone (PR) receptors.	[113]
hUCMSCs (2016)	Rat	The transplantation of hUCMSCs reduced the fibrosis area of endometrium, and concomitantly enhance glandular count and upgrade proliferation of the respective cells.	[114]
eMSCs (2014)	Human	Clonogenic Sushi Domain containing 2 and eMSCs were detected in the regenerated endometrium, endometrium Mesenchymal Stem Cell (eMSC) providing a viable alternative origin of MSC for future use within cell-based approaches.	[115]
hESCS (2014)	Rat	The mesenchymal origin of human Endometrial Side Population (hESP) was certified by their degree of specialization in vitro into osteocytes and adipocytes. After transplantation under renal capsule of NOS-SCID mice, where a potency in terms of human endometrium generation in rodents was highlighted.	[116]
CD45 + HPS (2007)	Mouse	Through this study performed on a novel transgenic mouse was demonstrated that CD45-positive HPS migrate to the uterine epithelium, forming approximately 80% of the epithelium during gestation.	[117]
Endometrial Side population (2010)	Mice	Following the xenotransplantation under the kidney capsule of NOG mice, unfractioned single-cell suspensions of endometrium derived from hysterectomy tissue boost the generation of the endometrial tissue.	[118]
hESCS (2014)	Rat	According to authors results, hESCs in parallel with collagen scaffolds could be notably supported the function recovery and uterine repair in a rodent model of intense uterine injury.	[119]
BMSCs (2014)	Rat	BMSCs can play a crucial role in thin endometrium reconstruction, division and immunomodulatory.	[120]

**Table 5:** Approaches dedicated to treating the Ahserman syndrome.

Treatment	Dilatation and Curettage Hysterescopy Hysteretomy
Re-adhesion prevention	Intrauterine Device Uterine balloon stent Foley’s catheter Anti-adhesions barriers Hyaluronic gel
Restoration of the endometrium	Hormonal treatment Stem cells
Post-operative assessment	Diagnostic hysteroscopy Ultrasound
